# New architectural design of delivery room reduces morbidity in preterm neonates: a prospective cohort study

**DOI:** 10.1186/s12884-016-0849-4

**Published:** 2016-03-23

**Authors:** Gianluca Terrin, Francesca Conte, Antonella Scipione, Vincenzo Aleandri, Maria Di Chiara, Erica Bacchio, Francesco Messina, Mario De Curtis

**Affiliations:** Department of Gynecology-Obstetrics and Perinatal Medicine, “Sapienza” University of Rome, Viale del Policlinico 155, 00161 Rome, Italy; Department of Pediatrics, “Sapienza” University of Rome, Viale del Policlinico 155, Rome, 00161 Italy; Research Center on Evaluation of Quality in Medicine - CEQUAM, “Sapienza” University of Rome, Rome, Italy; Department of Perinatal Medicine, Evangelical Hospital “V. Betania”, Via Argine 604, Naples, 80147 Italy

**Keywords:** Delivery room, Neonatal intensive care unit, Resuscitation, Hypothermia, Early nasal-CPAP, Morbidity, Intraventricular hemorrhage, Patent ductus arteriosus, Sepsis

## Abstract

**Background:**

A multidisciplinary committee composed of a panel of experts, including a member of the American Academy of Pediatrics and American Institute of Architects, has suggested that the delivery room (DR) and the neonatal intensive care units (NICU) room should be directly interconnected. We aimed to investigate the impact of the architectural design of the DR and the NICU on neonatal outcome.

**Methods:**

Two cohorts of preterm neonates born at < 32 weeks of gestational age, consecutively observed during 2 years, were compared prospectively before (Cohort 1: “conventional DR”) and after architectural renovation of the DR realized in accordance with specific standards (Cohort 2: “new concept of DR”). In Cohort 1, neonates were initially cared for a conventional resuscitation area, situated in the DR, and then transferred to the NICU, located on a separate floor of the same hospital. In Cohort 2 neonates were assisted at birth directly in the NICU room, which was directly connected to the DR via a pass-through door. The primary outcome of the study was morbidity, defined by the proportion of neonates with at least one complication of prematurity (i.e., late-onset sepsis, patent ductus arteriosus, intraventricular hemorrhage, periventricular leukomalacia, bronchopulmonary dysplasia, retinopathy of prematurity and necrotizing enterocolitis). Secondary outcomes were mortality and duration of hospitalization. Statistical analysis was performed using standard methods by SPSS software.

**Results:**

We enrolled 106 neonates (56 in Cohort 1 and 50 in Cohort 2). The main clinical and demographic characteristics of the 2cohorts were similar. Moderate hypothermia (body temperature ≤ 35.9 ° C) was more frequent in Cohort 1 (57 %) compared with Cohort 2 (24 %, *p* = 0.001). Morbidity was increased in Cohort 1 (73 %) compared with Cohort 2 (44 %, *p* = 0.002). No statistically significant differences in mortality and median duration of hospitalization were observed between the 2 cohorts of the study.

**Conclusions:**

If realized according to the proposed architectural standards, renovation of DR and NICU may represent an opportunity to reduce morbidity in preterm neonates.

## Background

The American Academy of Pediatrics (AAP) and American College of Obstetricians and Gynecologists have published several editions of their Guidelines for Perinatal Care [1], and The American Institute of Architects (AIA) has similarly published their Guidelines for Construction of Hospital and Healthcare Facilities [2]. More recently, a multidisciplinary committee, consisting of a panel of experts including a member of AAP and AIA, suggested a specific architectural design of the delivery room (DR) directly connected with neonatal intensive care unit (NICU) via a pass-through door, hypothesizing an improvement in the efficiency of preterm neonate stabilization procedures [3].

Commonly, care to neonates at birth is provided in area located within the DR and, only when necessary, after initial stabilization and a brief transportation, they continue in the NICU [4]. Although this architectural organization could be acceptable for healthy term and late-preterm neonates, it may not be appropriate for critical preterm neonates [3, 5]. Resuscitation and stabilization of these subjects would require the use of advanced and expensive devices, which are preferentially located in the NICU rooms [6]. In addition, the difficult stabilization process could be complicated by handling and moving in the first minutes of life [7–9]. For these reasons, the construction of a DR directly connected via pass-through doors to the NICU appears a more appropriate strategy to meet the needs of neonates and to optimize human and economic resources. To date, no study has verified the efficacy of different architectural organization on neonatal outcomes. Starting from these considerations, we aimed to evaluate the impact of architectural DR and NICU renovation performed in accordance to predefined standards [3] on neonatal morbidity, mortality and hospitalization time.

## Methods

We designed a prospective cohort study including inborn neonates, with gestational age < 32 weeks, consecutively observed in the NICU of Evangelical Hospital “V. Betania” of Naples, Italy, from 1 January 2008 to 31 December 2009. The study was carried out in compliance with Helsinki Declaration and the ethics committee (EC) of Evangelical Hospital “V. Betania” approved it (EC number: 15233). Written informed consent was obtained from the parents of the neonates enrolled in the study. Exclusion criteria were genetic syndromes, immunodeficiency, malformations and early onset sepsis. We enrolled neonates in two cohorts according to the architectural organization of NICU and DR in which they were assisted at birth. Specifically, neonates enrolled from 1 January 2008 to 31 March 2009 were initially cared in a conventional resuscitation area situated in the DR and then transferred to the NICU, located at separate floor of the same hospital (Cohort 1: period of “conventional DR”). Starting from 1 April 2009, the NICU underwent architectural renovation in accordance with recent standards defined for neonatal DR and NICU design [3]. Thus, from 1 April to 31 December 2009, neonates were born in a DR directly connected to NICU via a pass-through door (Cohort 2: period of “new concept of DR”). Equipment available for the initial stabilization of neonates was similar for the two cohorts of the study. In brief, self-inflating bag, T-piece resuscitator (Neopuff Infant Resuscitator, Fisher and Paykel, New Zealand), pulse-oximeter, humidifier, and oxygen blender were available for both cohorts of the study. Resuscitation was initially performed with an oxygen concentration ≤ 30 % and then titrated to maintain target saturation. Polyethylene plastic wrap was routinely used for babies delivered at less than 29 weeks gestation to avoid hypothermia. In the first period of the study (Cohort 1) after initial stabilization in the DR, neonates were transferred to the NICU by a neonatal incubator provided with a mechanical ventilator, without infant-flow system and heated and humidified gas. During the second study period (Cohort 2), respiratory support by invasive mechanical ventilation or by nasal continuous positive airway pressure (n-CPAP) with infant-flow system and heated and humidified gas, was started immediately after birth when appropriate, and it was not discontinued or modified if not specifically indicated by clinical needs. In NICU, standard care was applied in a similar manner, in both periods of the study, from recovery to discharge, according to our policies.

### Endpoint

The main endpoint was morbidity, defined by the proportion of neonates presenting at least one of the main complications of prematurity reported below: late-onset sepsis (LOS) [10, hemodynamically significant patent ductus arteriosus (PDA) [11], intraventricular hemorrhage (IVH) [12], periventricular leukomalacia (PVL) [13], bronchopulmonary dysplasia (BPD) [14], retinopathy of prematurity (ROP) [15], and necrotizing enterocolitis (NEC) [16]. We also compared the mortality rates and duration of hospitalization in the two cohorts of the study.

### Data collection

Clinical data regarding: birth weight, gestational age at birth, mode of delivery, multiple birth, sex, Apgar score at 1 and 5 min, antenatal corticosteroid treatment, indication for preterm delivery, characteristics of resuscitation interventions at birth, body temperature within the first 60 min of life, administration of exogenous surfactant, occurrence of LOS, PDA, IVH, PVL, BPD, ROP, and NEC were collected by researchers unaware of the study aims and design. Diagnosis of LOS, PDA, IVH, PVL, BPD, ROP, and NEC were performed according to previously used criteria [10–16]. Data on duration of hospital stay and mortality were also collected and analyzed.

## Statistics

To demonstrate a 20 % of reduction in morbidity between the two cohorts of the study with 80 % power and type error = 0.05 (2-tailed test), a minimum sample size of 50 neonates for each group was required. The Kolmogorov-Smirnov test was used to determine whether variables were normally distributed. For continuous variables, groups were compared by using the t test of equality of means, the Mann-Whitney *U* test, and the Kruskal-Wallis test. The chi-square test and Fisher’s exact test were used for categorical variables. We performed a multivariate analysis using binary logistic regression analysis to evaluate whether morbidity was influenced by confounding variables (i.e., gestational age, birth weight, mode of delivery, multiple birth, sex, antenatal corticosteroid treatment, Apgar score at 5 min, and study cohort assignment). The level of significance for all statistical tests was 2-sided (*p* < 0.05). Statistical analysis was performed by a statistician blinded to patient group assignment, using SPSS version 19.0 for Windows (SPSSInc).

## Results

We enrolled 106 neonates, 56 in Cohort 1 and 50 in Cohort 2. Main clinical and demographic characteristics of the two cohorts were similar, as reported in Table 1.

**Table 1:** Main clinical characteristics and resuscitation interventions at birth of study population

	Cohort 1 (conventional delivery room)^a^	Cohort 2 (new concept of delivery room)^a, b^	p
Number of neonates	56	50	-
Clinical characteristics			
Body birth weight, g	1114 ± 285	1033 ± 315	0.066
Gestational age, w	28 ± 0.3	28 ± 0.5	0.480
Caesarean section, n (%)	50 (89)	46 (92)	0.746
Twins, n (%)	14 (25)	18 (36)	0.192
Male, n (%)	29 (52)	22 (44)	0.423
Apgar at 1 min	5 ± 0.2	5 ± 0.1	0.850
Apgar at 5 min	7 ± 0.1	7 ± 0.2	0.430
Prenatal corticosteroids, n (%)	32 (58)	22 (44)	0.146
Surfactant therapy, n (%)	40 (71)	42 (84)	0.123
Indications for preterm delivery			
Spontaneous preterm labour	40	37	0.767
Preterm premature rupture of membranes	8	6	0.422
Hypertensive disorders of pregnancy	6	5	0.904
Intrauterine growth restriction	2	2	0.908
Resuscitation interventions at birth			
Oxygen therapy, n (%)	53 (95)	45 (90)	0.471
Positive pressure ventilation with face mask, n (%)	23 (41)	25 (50)	0.357
Intubation, n (%)	34 (61)	29 (58)	0.776
Drug administration, n (%)	1 (2)	2 (4)	0.610
Chest compression, n (%)	4 (7)	2 (4)	0.679
Nasal-CPAP in delivery room	10 (17.9)	9 (18.0)	0.985
Nasal-CPAP without interruption during the first 30’ after birth^c^	0 (0.0)	9 (18)	0.001

^a^Data expressed as mean ± standard deviation, when not specified

^b^DR directly connected to the NICU according to architectural standards, as described in the text.

^c^In the Cohort 1 nasal-CPAP administered by infant-flow system with heated and humidified gas, started in delivery room, was discontinued during transportation from point of delivery to the neonatal intensive care unit (NICU) located at different floor of the same Hospital. During the transportation to the NICU, neonates in the Cohort 1, were ventilated with nasal-CPAP without infant-flow system, heated and humidified gas

**Fig. 1:**
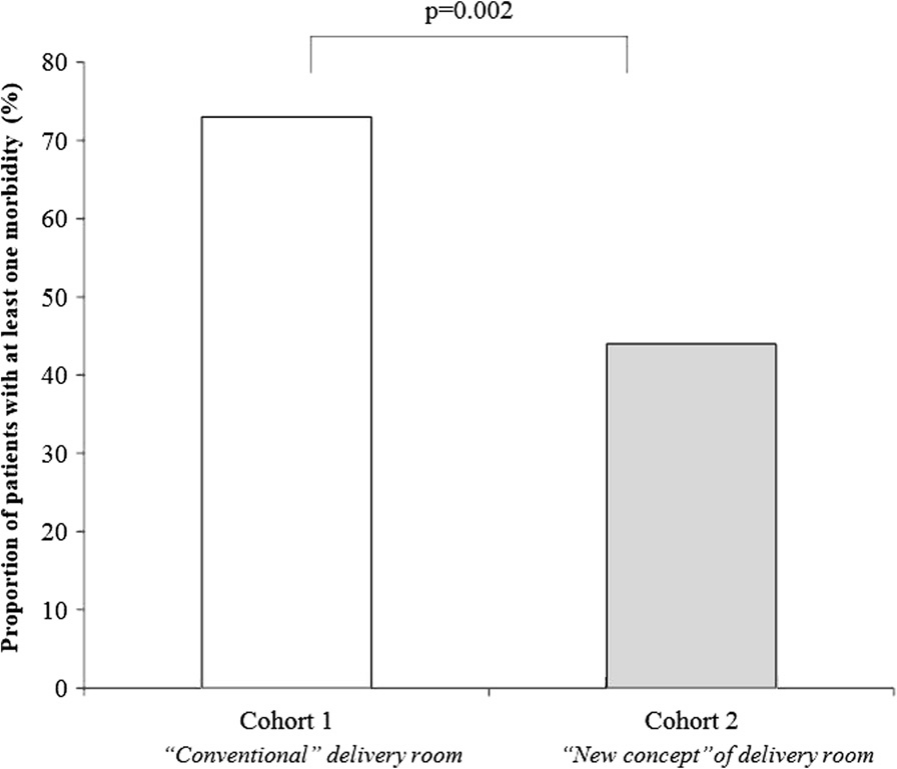
Influence of Architectural design of delivery room on morbidity

Morbidity was increased in Cohort 1 compared with Cohort 2 (Fig. 1). In particular, the rate of neonates presenting LOS, PDA and IVH (≥ II grade) was significantly increased in Cohort 1 compared with Cohort 2 (Table 2). Occurrence of moderate hypothermia defined as body temperature ≤ 35.9 ° C [17] within the first 60 min of life was more prevalent in Cohort 1 (57.1 %) compared with Cohort 2 (24.0 %, *p* = 0.001).

**Table 2:** Main outcomes of study population

	Cohort 1 (conventional DR)	Cohort 2 (new concept of DR^a^)	p
Number of neonates	56	50	-
Outcomes			
Late-onset sepsis, n (%)	10 (18)	1 (2)	0.008
Late-onset culture-proven sepsis, n (%)	7 (12.5)	1 (2.0)	0.043
Patent ductus arteriosus^b^, n (%)	26 (46)	13 (26)	0.029
Intraventricular hemorrhage, n (%)	19 (34)	6 (12)	0.008
Intraventricular hemorrhage ≥ II grade, n (%)	9 (16)	2 (4)	0.042
Intraventricular hemorrhage ≥ III grade, n (%)	3 (5.4)	2 (4.0)	0.554
Periventricular leukomalacia, n (%)	2 (4)	3 (6)	0.556
Necrotizing enterocolitis, n (%)	1 (2)	3 (6)	0.341
Bronchopulmonary dysplasia^c^, n (%)	9 (19)	7 (15)	0.649
Retinopathy of prematurity, n (%)	7 (12)	6 (12)	0.938
Exitus, n (%)	8 (14)	5 (10)	0.502

^a^DR directly connected to the NICU according to architectural standards, as described in the text.

^b^Hemodynamically-significant patent ductus arteriosus requiring a pharmacologic treatment, as described in the text.

^c^Defined as need of oxygen therapy at 36 weeks of post-conceptional age

The use of non-invasive ventilation without interruption during the first 30 min of life was higher for neonates included in Cohort 2 (Table 2). No difference in mortality rate was observed between the two study cohorts (14.3 % in Cohort 1 vs 10.0 % in Cohort 2, *p* = 0.502). The median duration of hospitalization for survivals was similar in the two study cohorts (51 days, interquartile range 36, in Cohort 1 *vs*. 53 days, interquartile range 34, in Cohort 2, *p* = 0.541). A multivariate analysis demonstrated that morbidity was associated with body birth weight and by cohort assignment (Table 3).

**Table 3:** Multivariate analysis evaluating the influence of different variables on morbidity

Variables	B	Odds ratio (95 % confidence interval)	p
Birth weight	‐ 0.002	0. 998 (0.996 - 1.000)	0.023
Architectural design of DR^a^	- 1.457	0.233 (0.089 - 0.609)	0.003
Prenatal corticosteroids	- 0.336	0.714 (0.280 - 1.825)	0.482
Twin pregnancy	0.128	1.136 (0.412 - 3.138)	0.805
Cesarean section	‐ 0.000	1.000 (0.154 – 6.478)	1.000
5’ Apgar score	- 0. 284	0.753 (0.438 – 1.296)	0.306

^a^new architectural design reduce risk of morbidity in a multivariate analysis constant 5372

## Discussion

The main goal of the architectural organization of a DR is to facilitate the transition of the fetus to the extra- uterine life [18]. Hospitals vary in their approach to the details of how to prepare the resuscitation area for neonates at birth. Some hospitals have an area of the DR equipped for neonatal resuscitation not in direct communication with the NICU, whereas others have the DR adjacent to the NICU, allowing assistance of critical neonates directly in the environment of the intensive care unit [4, 18]. Previous studies analyzed how different clinical practices at birth may influence preterm neonatal health [18]. However, the impact of different architectural designs of DR on clinical outcomes had not been previously verified. Recent data showed that up to 80 % of delivery services resuscitate newborns in the DR, while less than 8 % admit infants directly into a resuscitation bed in NICU adjacent to DR [4]. We demonstrated that the last option may have significant advantages for preterm neonates. Optimization of assistance in the first minutes of life would help to improve transition from fetal to neonatal life, reducing the risk of destabilization, particularly in preterm neonates [6]. However, recommendations for resuscitation of preterm neonates at birth are not respected in many points [4, 19–21]. Discrepancies between recommendations and clinical practice may depend, at least in part, on the inadequacy of areas dedicated to resuscitation at birth [4, 19–23]. It has been suggested that the incorporation of an intensive care environment into the DR could promote the application of adequate resuscitation practice at birth, potentially leading to a reduction of early and long-term complications of prematurity [6, 9]. If this organization is desirable, the realization of the resuscitation area in duplicate (i.e. in DR and in NICU) in the same hospital is expensive in terms of both economic and human resources. In addition, it has been widely reported that morbidity and mortality rates are increased for the most immature neonates who require transportation [24–26]. Our results suggest that even brief transportations from DR to NICU between different points of the same hospital may have negative consequences on neonatal outcomes. On the basis of these considerations, a DR in direct communication with the NICU probably represents the most appropriate architectural solution [3].

The positive effects observed in neonates receiving the first care directly in the NICU may have multiple explanations. Firstly, the reduction of the risk of hypothermia, considered a crucial aspect of resuscitation at birth [27]. Despite advances in techniques adopted to maintain the newborn infant’s temperature within the normal range during stabilization (i.e., radiant warmers, heated mattress, polyethylene bag and/or cap, increasing the DR temperature), a significant rate of hypothermia was frequently reported [28–33]. The neonates who require transportation during the first minute of life often cross into and out of multiple different environments, with wide temperature and humidity variations and consequent increased risk of hypoxemia and increased pulmonary resistance [8]. Hypothermia and re-warming have been also associated with increased activity of prostaglandin-synthase and nitric oxide synthase, that are crucial mediators in the pathogenesis of this complication of prematurity, including persistence of ductus arteriosus [34, 35]. In a large observational study, up to 46 % of very low birth weight (VLBW) neonates were moderately hypothermic at admission in NICU [29]. Laptook et al. also demonstrated that the admission temperature was inversely related to mortality and LOS rate, with 28 % increase in mortality and 11 % increase in LOS per 1 °C of decrease in body temperature [29]. Similarly, Miller et al. found that mild and moderate hypothermia were very common among VLBW neonates admitted in NICU and they revealed a positive association between moderate hypothermia and adverse neonatal outcomes [33]. Accordingly, we confirmed that maintenance of optimal body temperature is essential to reduce hypothermia and related morbidities [29, 33, 36]. For the first time, we have demonstrated that this objective may be facilitated when neonates are assisted directly in a NICU at birth [3, 37].

The early use of n-CPAP at birth has been recommended by recent guidelines in order to promote alveolar recruitment [38–40]. At the same time, alveolar de-recruitment may have a negative impact on neonatal stabilization [41, 42]. In Cohort 2 the n-CPAP was administered by using an infant-flow system, heated and humidified gas, without any interruption during stabilization period, and thus without risk of alveolar de- recruitment [41, 42]. In Cohort 1, the use of infant-flow n-CPAP system and of heated and umidified gas was interrupted during transportation from the point of delivery to the NICU. We speculate that this interruption may have determined alveolar de-recruitment and additional loss of body temperature and possible increase in pulmonary resistance [36, 41, 42].

Although interesting, the results of this study should be interpreted considering its limitations. The study has a non-randomized design. However, for obvious ethical reasons it is difficult to address the aim of the research with a different study design. The high rate of LOS and of IVH observed in the Cohort 1 may depend on the small number of the patients enrolled in each study period. Thus, the differences in these outcomes between the two cohorts, despite their statistical significance, should be interpreted with caution, because they may be coincidental rather than dependent on architectural renovation. Finally, the limited use of prenatal steroids may have influenced final neonatal outcomes. These results may be secondary to the high number of mothers not receiving adequate prenatal care and who were directed to the birth-Hospital only when preterm delivery was imminent. However, the use of prenatal steroids was similar between the two cohorts of the study.

## Conclusions

Architectural renovation of DR may represent an opportunity to improve the outcome of preterm babies, if performed according to specific standards [3]. Despite the necessity of further evidence, this study provides useful information for planning architectural organization of the DR. The optimization of human and instrumental resources obtained with this kind of intervention merits further research.

## Abbreviations

BPD: bronchopulmonary dysplasia
DR: delivery room
IVH: intraventricular hemorrhage
LOS: late onset 1 sepsis
n-CPAP: nasal continuous positive airway pressure
NEC: necrotizing enterocolitis
NICU: neonatal intensive care unit
PDA: patent ductus arteriosus
PVL: periventricular leukomalacia
ROP: retinopathy of prematurity
VLBW: very low birth weight
